# Video Super-Resolution Method Using Deformable Convolution-Based Alignment Network

**DOI:** 10.3390/s22218476

**Published:** 2022-11-03

**Authors:** Yooho Lee, Sukhee Cho, Dongsan Jun

**Affiliations:** 1 Department of Computer Engineering, Dong-A University, Busan 49315, Korea; 2 Media Intelligence Laboratory Electronics and Telecommunications Research Institute (ETRI), Daejeon 34129, Korea

**Keywords:** video super-resolution, convolutional neural network, alignment network, deformable convolution, dilated convolution, spatial attention, channel attention

## Abstract

With the advancement of sensors, image and video processing have developed for use in the visual sensing area. Among them, video super-resolution (VSR) aims to reconstruct high-resolution sequences from low-resolution sequences. To use consecutive contexts within a low-resolution sequence, VSR learns the spatial and temporal characteristics of multiple frames of the low-resolution sequence. As one of the convolutional neural network-based VSR methods, we propose a deformable convolution-based alignment network (DCAN) to generate scaled high-resolution sequences with quadruple the size of the low-resolution sequences. The proposed method consists of a feature extraction block, two different alignment blocks that use deformable convolution, and an up-sampling block. Experimental results show that the proposed DCAN achieved better performances in both the peak signal-to-noise ratio and structural similarity index measure than the compared methods. The proposed DCAN significantly reduces the network complexities, such as the number of network parameters, the total memory, and the inference speed, compared with the latest method.

## 1. Introduction

Sensors are used in a wide range of fields, such as autonomous driving, robotics, Internet of Things, medical, satellite, military, and surveillance. The development of sensors leads to miniaturization and increased performance. Image and video sensors are essentially used to handle the visual aspect. Although image and video sensors were developed to work in environments of low latency and complexity, they operated in environments with low network bandwidth, which limits the quality of input images and videos. Therefore, various image and video processing methods, such as super-resolution (SR) [1,2,3,4,5,6,7,8], deblurring [9,10,11,12,13], and denoising [14,15,16,17], are used for restoration.

SR aims to generate high-resolution (HR) data from low-resolution (LR) data. Despite the initial SR methods based on pixel-wise interpolation algorithms, such as bicubic, bilinear, and nearest neighbor, being straightforward and intuitive in strategy, they have limitations in reconstructing high-frequency textures in the interpolated HR area.

With the development of deep learning technologies, image or video SR methods are currently investigated using convolutional neural network (CNN) [18] and recurrent neural network (RNN) [19]. Although deep learning-based SR methods [20,21,22,23,24,25,26,27,28,29,30,31,32,33] have superior performance, with development, parameter size and memory capacity are increased in the networks. Thus, methods for reducing network complexity are proposed for use in sensors of lightweight memory and limited computing environment devices such as smartphones.

In this paper, we propose a deformable convolution-based alignment network (DCAN) with a lightweight structure, which enhances perceptual quality better than the previous methods in terms of peak signal-to-noise ratio (PSNR) [34] and structural similarity index measure (SSIM) [35]. Through a variety of ablation studies, we also investigate the trade-off between the network complexity and the video super-resolution (VSR) performance in optimizing the proposed network. The contributions of this study are summarized as follows:

- To improve VSR performance, we propose two alignment blocks designed to combine dilation and attention-based deformable convolution and develop two alignment methods using the neighboring input frames, such as attention-based alignment block (AAB) and dilation-based alignment block (DAB), in the proposed VSR model. Firstly, AAB extracts characteristics similar to the current frame using the attention method to obtain spatial and channel weights using max and average pooling. Secondly, DAB learns a wide range of receptive fields of feature maps by applying dilated convolution.
- Through the optimization for our model, we conducted a tool-off test on AAB and DAB, Resblock in the alignment block and up-sampling block, and the pixel-shuffle layer. Firstly, AAB and DAB increased SR performance by 0.64 dB. Secondly, optimal Resblock in the alignment block and up-sampling block enhanced SR performance by 0.5 and 0.73 dB, respectively. Thirdly, the model using two pixel-shuffle layers was better than the model using one layer, by 0.01 dB.
- Finally, we verified that the proposed network can improve PSNR and SSIM by up to 0.28 dB and 0.015 on average, respectively, compared to the latest method. The proposed method can significantly decrease the number of parameters, total memory size, and inference speed by 14.35%, 3.29%, and 8.87%, respectively.

The remainder of this paper is organized as follows: In Section 2, we review the previous CNN-based VSR methods, including the essential network components. In Section 3, we describe the frameworks of the proposed DCAN. Finally, experimental results and conclusions are presented in Section 4 and Section 5, respectively.

## 2. Related Works

Although pixel-wise interpolation methods were conventionally used in initial SR, it was difficult to properly represent the complex textures with high quality in the interpolated SR output. As CNN-based approaches have recently produced convincing results in the image and video restoration area, SR methods that use CNN can also achieve more SR accuracy than the conventional SR methods.

**Figure 1:**
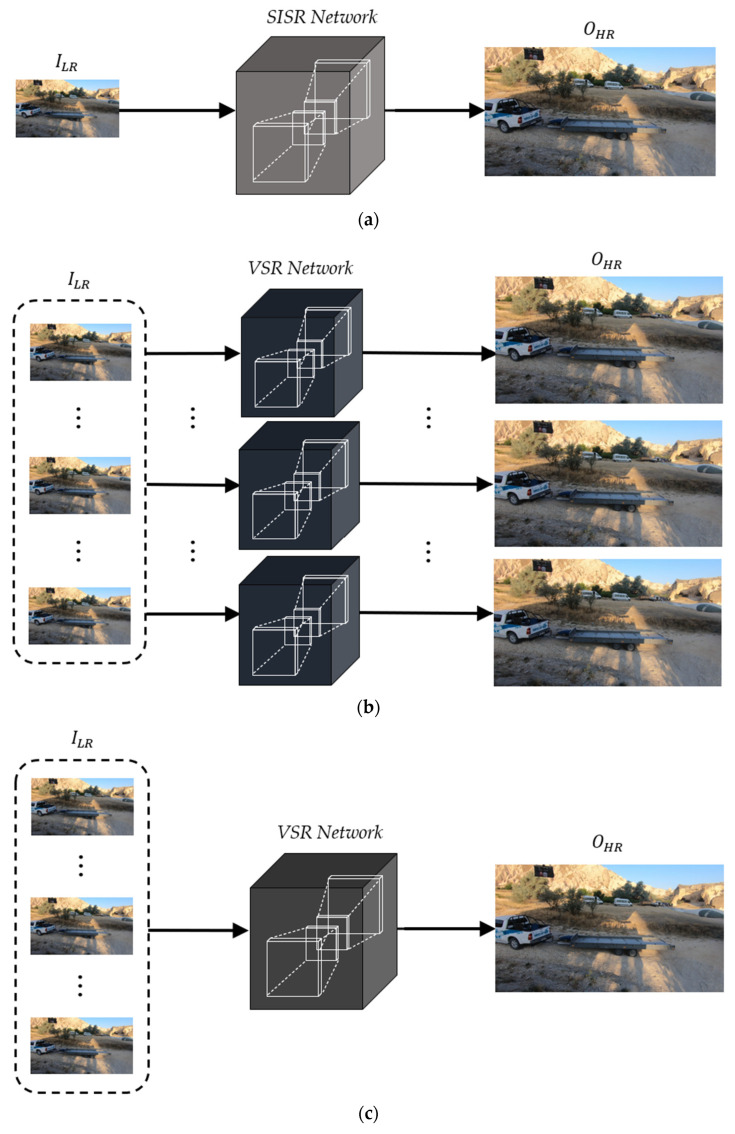
CNN-based image and video super-resolution schemes. (**a**) Single-image SR (SISR), (**b**) video SR (VSR) to generate multiple high-resolution frames, and (**c**) VSR to generate a single high-resolution frame.

Figure 1 shows the CNN-based image and video super-resolution schemes. Figure 1a is the general architecture of a single-image super-resolution (SISR) to generate an HR image ($O_{HR}$) from an LR image ($I_{LR}$). On the other hand, most video super-resolution (VSR) methods generate multiple HR frames from the corresponding LR frames, as shown in Figure 1b. Although these approaches can be implemented with simple and intuitive network architectures, they tend to degrade the VSR performance due to a lack of temporal correlations between consecutive LR frames.

To overcome the limitations of the previous VSR schemes, recent VSR methods have been designed to generate single HR frames from multiple LR frames, as shown in Figure 1c. Note that the generated single HR frame corresponds to the current LR frame. To improve the VSR performance in this approach, it is important that the neighboring LR frames be aligned to contain as much context of the current LR frame as possible before conducting CNN operations at the stage of input feature extraction. As one of the alignment methods, optical flow can be applied to each neighboring LR frame to perform pixel-level prediction through the two-dimensional (2D) pixel adjustment.

Although this scheme can provide better VSR performance compared to that of the conventional VSR schemes, as in Figure 1b, all input LR frames including the aligned neighboring frames are generally used with the same weights. It means that the VSR network generates a single HR frame without considering the priorities between them. In addition, the alignment processes generally make the VSR networks more complicated due to the increase in total memory size and number of parameters.

The exponential increase in GPU performance has enabled the development of more sophisticated networks with deeper and denser CNN architectures. To design elaborate networks, there are several principal techniques to extract more accurate feature maps in the process of convolution operations, such as spatial attention [36], channel attention [37], dilated convolution [38], and deformable convolution [39].

**Figure 2:**
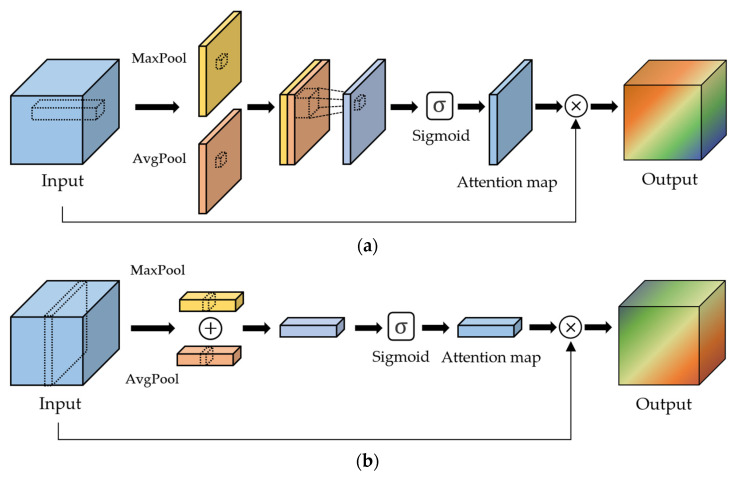
Spatial and channel attention to assign different priorities of input feature maps. (**a**) Spatial attention and (**b**) channel attention.

Spatial attention: Spatial attention improves the accuracy of the feature maps. As shown in Figure 2a, it generates a spatial attention map after combining the intermediate feature maps from max and average pooling. Note that the spatial attention map consists of weight values between 0 and 1 as the result of the sigmoid function. Then, all features in the same location over the channels of the intermediate feature maps are multiplied by the corresponding weight of the spatial attention map.

Channel attention: The aim of channel attention is to allocate different priorities to each channel of the feature maps generated by convolution operations. Initial channel attention was proposed by Hu et al. [37] in the squeeze-and-excitation network (SENet). Like spatial attention, Woo et al. [36] proposed to generate a channel attention map using max and average pooling per each channel, as shown in Figure 2b. Then, each channel of the feature maps is multiplied by the corresponding weight of the channel attention map.

**Figure 3:**
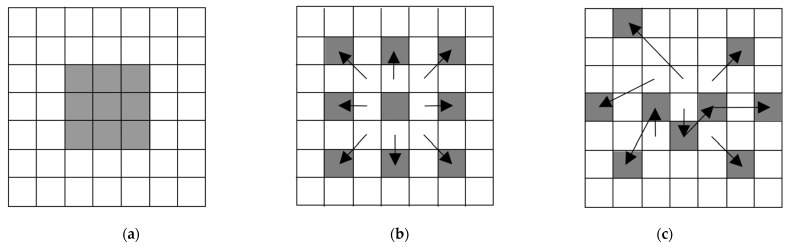
Examples of various convolution operations where the kernel is marked as gray pixels and its size is 3 × 3. (**a**) Conventional convolution. (**b**) Dilated convolution with dilation factor 2. (**c**) Deformable convolution.

Dilated convolution: While convolution operations with the different multiple kernels can generally extract better output feature maps, it requires an extra burden, such as the increase of the kernel parameters. The aim of dilated convolution is to have similar effects with the different multiple kernels while reducing the number of kernel parameters. In Figure 3a, it means that dilation factor 1 is equivalent to the conventional convolution. On the other hand, convolution operations are applied to the 5 × 5 input feature area according to the number of dilation factors, as shown in Figure 3b.

Deformable convolution: In terms of neural network-based tasks, motion is adaptively adjusted through deformable convolution [39], optical flow [40], and motion attentive [41] methods. To obtain better output features, the deformable convolution helps to find the exactly matched input feature corresponding to each kernel parameter. Contrary to the conventional operation, it generates two feature maps, which indicate X and Y axis offsets to shift the kernel parameter for geometric transformations, as shown in Figure 3c. Although deformable convolution using multiple offsets [42] recently improved SR performance, the operation tends to be more complicated, with huge parameter sizes and memory consumption.

With the mentioned techniques, various VSR networks have been designed to achieve better VSR performance. As the first CNN-based VSR method, Liao et al. [43] proposed the deep draft-ensemble learning (Deep-DE) architecture, which was composed of three convolution layers and a single deconvolution layer. Since the advent of Deep-DE, Kappeler et al. [44] proposed a more complicated VSR network (VSRnet), which consists of motion estimation and compensation modules to align the neighboring LR frames and three convolution layers, with the rectified linear unit (ReLU) [45] used as an activation function. Caballero et al. [46] developed a video-efficient sub-pixel convolution network (VESPCN) to effectively exploit temporal correlations between the input LR frames. It also adopted a spatial motion compensation transformer module to perform the motion estimation and compensation. After the feature maps are extracted from the motion-compensated input frames, an output HR frame is generated from them using a sub-pixel convolution layer. Jo et al. [47] proposed dynamic up-sampling filters (DUF), which consist of 3D convolution filters to replace motion estimation, dynamic filter, and residual learning.

Isobe et al. [48] developed the temporal group attention (TGA) structure to fuse spatio-temporal information through the frame-rate-aware groups hierarchically. It introduced a fast spatial alignment method to handle input LR sequence videos with large motion. Additionally, TGA adopted 3D and 2D dense layers to improve SR accuracy. As feature maps generated by previous convolution operations are concatenated with the current feature maps, it demands a large parameter size and memory. In the super-resolve optical flows (SOF) for the video super-resolution network [49], it was composed of an optical flow reconstruction network, motion compensation module, and SR network to exploit the temporal dependency. Although optical flows for the video super-resolution network improved VSR performance by recovering temporal details, this type of approach caused a kind of blurring effect due to the excessive motion compensation. In addition, it used down-sampling and up-sampling at each level and caused a loss in the feature map information. Tian et al. [50] proposed a temporally deformable alignment network (TDAN), which was designed with multiple residual blocks and a deformable convolution layer. As it lacked preprocessing before the deformable convolution operation, it had limitations in improving the SR accuracy of the generated HR frame. Wen et al. [51] proposed a spatio-temporal alignment network (STAN) which consists of a filter-adaptive alignment network and an HR image reconstruction network. After the iterative spatio-temporal learning scheme of the filter adaptive alignment network extracts the intermediate feature maps from the input LR frames, a final HR frame is generated using the HR image reconstruction network, which consists of twenty residual channel attention blocks and two up-sampling layers. Although STAN achieved higher VSR performance than the previous methods, its limitation is in feature alignment of the corresponding current frame repeatably using the aligned feature maps of the corresponding previous frame. Besides, using hundreds of convolutions in the HR image reconstruction network, the number of parameters, memory size, and complexity were significantly increased.

**Figure 4:**
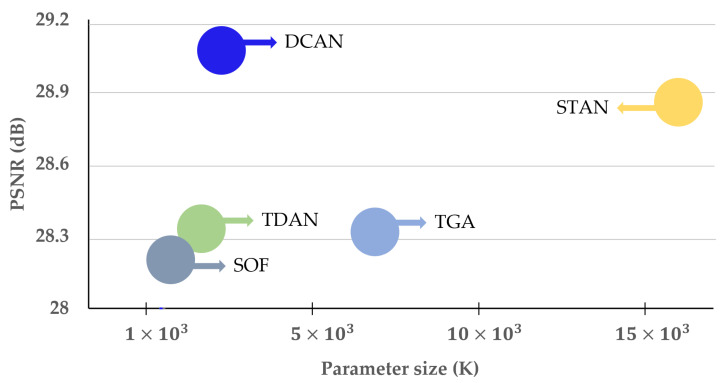
Comparison of network SR performance and complexity between the proposed DCAN and previous methods for the REDS4 test dataset. The x- and y-axes denote the parameter size and PSNR, respectively.

In this study, we designed the proposed method by supplementing the limitations of the previous method. Therefore, by learning the aligned current frame with the neighboring frame, as shown in Figure 4, our proposed method provides superior SR performance and is lightweight compared to the previous methods.

## 3. Proposed Method

### 3.1. Overall Architecture of DCAN

**Figure 5:**
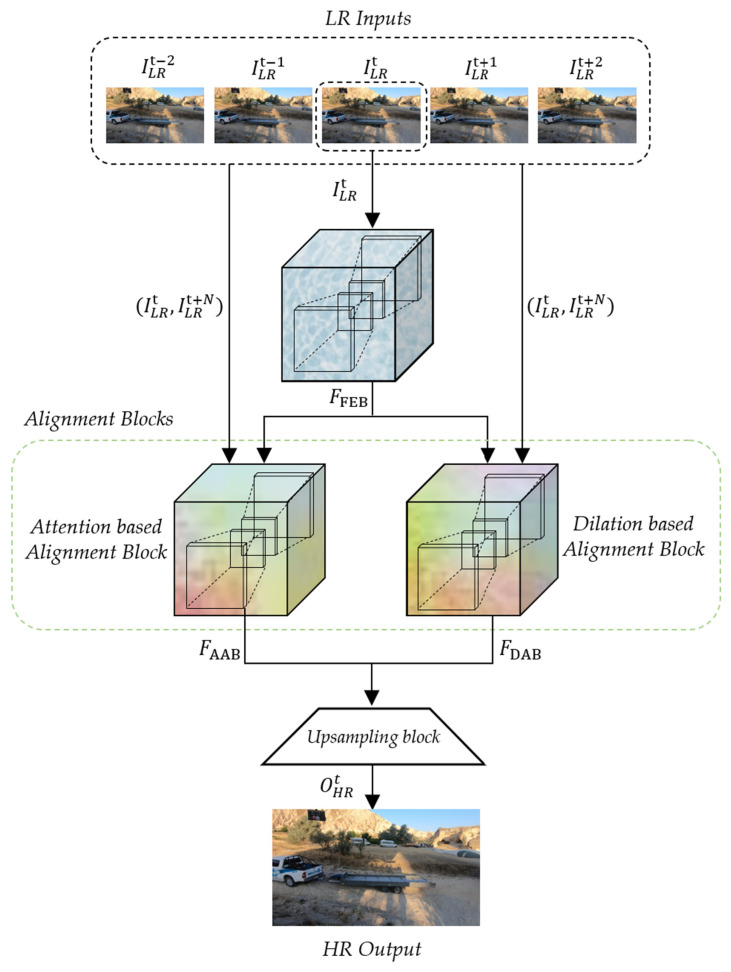
Overall architecture of the proposed DCAN.

The proposed deformable convolution-based alignment network (DCAN) generates a scaled HR sequence that is quadruple the size of the input LR sequence. As depicted in Figure 5, the proposed DCAN consists of a feature extraction block (FEB), two different alignment blocks to exploit the consecutive contexts between the neighboring LR frames, and an up-sampling block. In detail, the alignment blocks of DCAN are composed of AAB and DAB, which are commonly coupled with deformable convolution.

The input and output of DCAN are the five consecutive frames ($I_{LR}^{t+N},\;N=\left[-2:2\right]$) of the input LR sequence and the single reconstructed HR frame ($O_{HR}^{t}$), respectively. In this paper, the output feature maps of the $i^{th}$ convolution layer ($H_{C})$ are denoted as $F_{i}$ and they are computed as in Equation (1):

(1) $$F_{i}=H_{C}\left(F_{i-1}\right)=\sigma \left(W_{i}⊗F_{i-1}+B_{i}\right)$$

where $H_{c}^{i},\;\sigma \left(\cdot \right),\;W_{i},\;‘⊗’,\;\mathrm{and}\;B_{i}$ are denoted as the convolution operation of the $i^{th}$ layer with the parametric ReLU (PReLU) [52], the activation function, kernel weights, the weighted sum between the previous feature maps and kernel’s weights, and the biases of the kernels, respectively. The proposed DCAN uniformly sets the channel depth of the feature maps and kernel size as 64 and 3 × 3, respectively.

**Figure 6:**
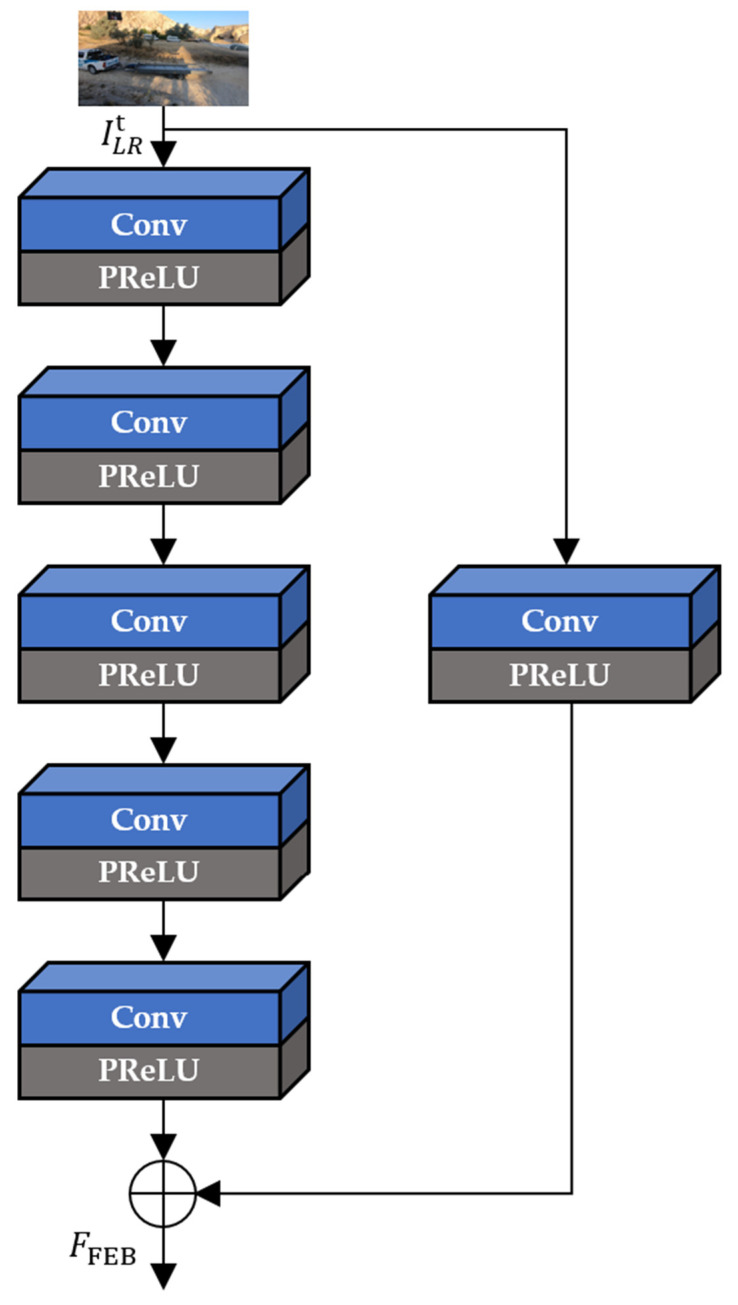
The architecture of FEB.

In Figure 6, FEB extracts the intermediate feature maps ($F_{FEB})$ from only the current input LR frame ($I_{LR}^{t}$) through the five iterative convolution operations. In addition, FEB performs the global skip connection to learn residual features and avoid the gradient vanishing effects, as in Equation (2):

(2) $$F_{FEB}=H_{C}^{5}\left(H_{C}^{4}\left(H_{C}^{3}\left(H_{C}^{2}\left(H_{C}^{1}\left(I_{LR}^{t}\right)\right)\right)\right)\right)+H_{C}(I_{LR}^{t}).$$

**Figure 7:**
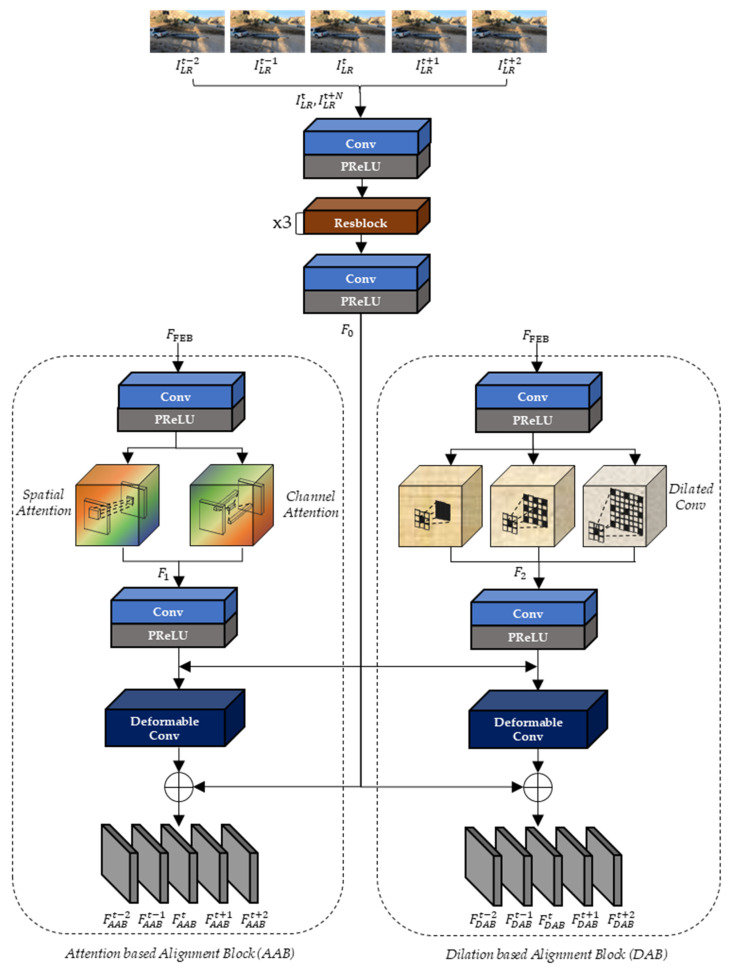
The architecture of alignment blocks.

**Figure 8:**
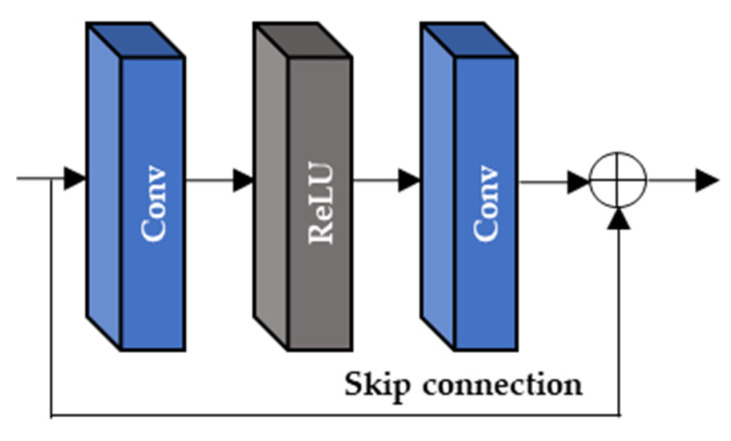
The Resblock of the proposed DCAN.

As depicted in Figure 7, the extracted feature maps, $F_{FEB}$, and two input LR frames, ($I_{LR}^{t},\;I_{LR}^{t+N}$), are commonly used as the inputs of the two alignment blocks (AAB and DAB). Since the range of N is from −2 to 2 in the input LR frame ($I_{LR}^{t+N}$), the 5 output feature maps of AAB and DAB ($F_{AAB}$ and $F_{DAB})$ are sequentially generated and they are corresponding to the $I_{LR}^{t+N}$. In the proposed DCAN, both AAB and DAB deploy Resblock of Figure 8 ($H_{R})$ and the deformable convolution ($H_{D}).$

In Figure 7, $F_{0}$, $F_{1}$, and $F_{2}$ are generated from the two input LR frames ($I_{LR}^{t},\;I_{LR}^{t+N}$), the spatial and channel attention of AAB, and three different dilated convolutions of DAB, respectively, as in Equations (3)–(5):

(3) $$F_{0}^{t+N}=H_{C}\left(H_{R}\left(H_{R}\left(H_{R}\left(H_{C}\left(I_{LR}^{t},\;I_{LR}^{t+N}\right)\right)\right)\right)\right),\;N=-2,-1,...\;2,$$

(4) $$F_{1}=[H_{SA}\left(H_{C}\left(F_{FEB}\right)\right),H_{CA}\left(H_{C}\left(F_{FEB}\right)\right)],$$

(5) $$F_{2}=[H_{df1}\left(H_{C}\left(F_{FEB}\right)\right),H_{df2}\left(H_{C}\left(F_{FEB}\right)\right),\;H_{df3}\left(H_{C}\left(F_{FEB}\right)\right)],$$

where $H_{SA}$, $H_{CA}$, $H_{df}$, and [·] perform the spatial attention, the channel attention, the dilated convolution with the dilation factors 1, 2, and 3, and concatenation, respectively.

The output feature maps ($F_{AAB})$ of AAB are sequentially generated from the input feature maps ($F_{1}$, $F_{0}^{t+N}$), as in Equation (6):

(6) $$F_{AAB}^{t+N}=H_{D}\left(H_{C}\left(F_{1}\right)+F_{0}^{t+N}\right)+F_{0}^{t+N},\;N=-2,-1,...\;2.$$

To use multiple kernels while reducing the number of kernel parameters, DAB adopts three dilated convolutions with dilation factors of two and three, which correspond to the wider kernel size (5 × 5 and 7 × 7). DAB generates the output feature maps ($F_{DAB}$), as in Equation (7):

(7) $$F_{DAB}^{t+N}=H_{D}\left(H_{C}\left(F_{2}\right)+F_{0}^{t+N}\right)+F_{0}^{t+N},\;N=-2,-1,...\;2.$$

In the alignment block, AAB can extract similar characteristics to the current frame by adopting the attention method to obtain spatial and channel weights using max and average pooling. Furthermore, DAB can learn a wide range of the receptive field of feature maps by applying dilated convolution. Therefore, unlike previous methods [48,49,50,51] that intuitively use input feature map characteristics before alignment, DCAN extracts the aligned current frame using deformable convolution after preprocessing with dilated convolution and attention methods.

**Figure 9:**
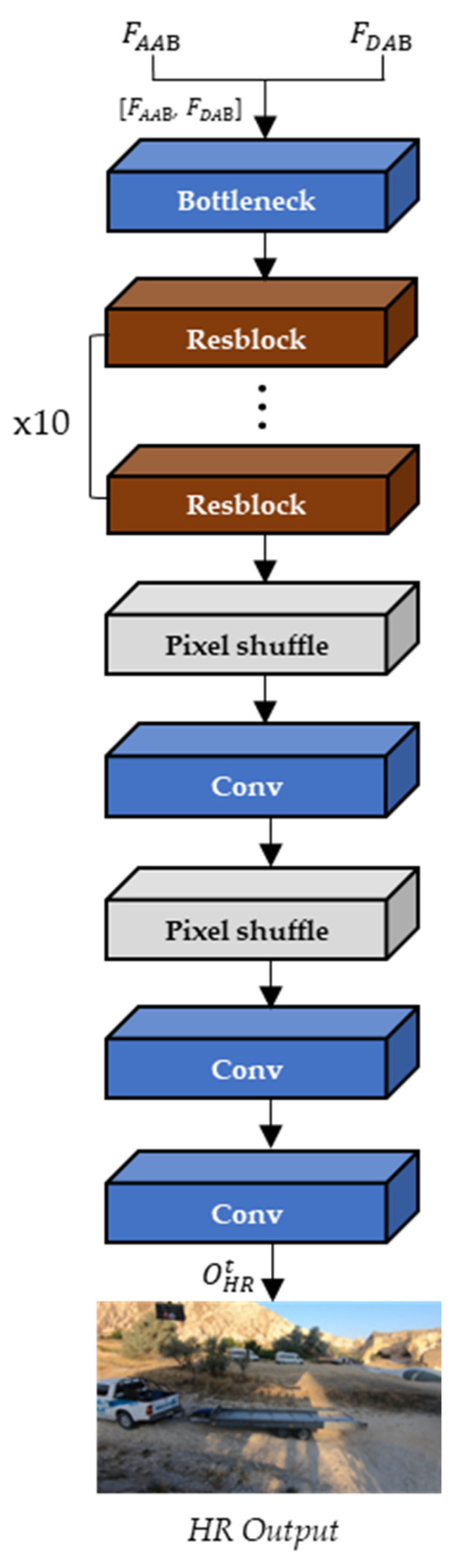
The architecture of the up-sampling block.

Then, the final output frame is generated from the up-sampling block with the concatenated $F_{AAB}$ and $F_{DAB}$. As shown in Figure 9, the upsampling block consists of one bottleneck layer to reduce the channel depth, ten Resblock, three convolution layers, and two pixel-shuffle layers to expand the spatial resolution of the input frames.

### 3.2. Ablation Works

**Figure 10:**
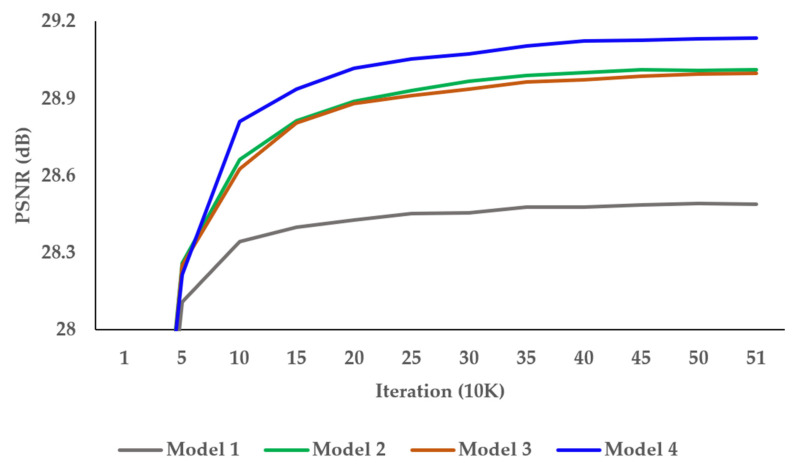
Investigations of the alignment block.

To find the optimal network architecture of the proposed DCAN, we conducted a tool-off test on the AAB and DAB in Table 1. As presented in Table 1, Model 1 showed the lowest performance without AAB and DAB. Model 2 had DAB added and achieved an enhancement of 0.5 dB over Model 1. Model 3 had AAB added and improved by 0.52 dB over Model 1. Although Model 2 and Model 3 performances differed insignificantly, AAB affected the performance more than DAB. Figure 10 shows the PSNR result per iteration of the tool-off test on AAB and DAB. It demonstrates well-trained results of DCAN without overfitting problems.

**Figure 11:**
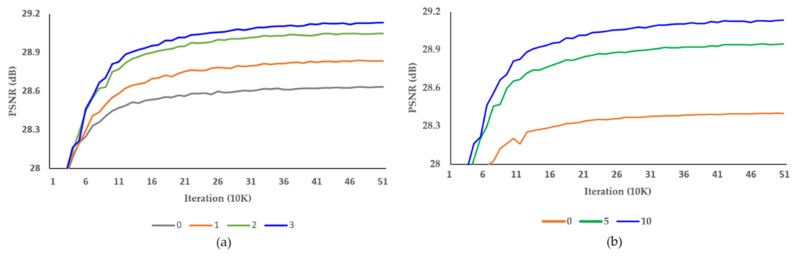
Investigation of the number of Resblocks in the (**a**) alignment and (**b**) up-sampling blocks, respectively. PSNR per iteration on REDS4 dataset.

Table 2 and Table 3 show the results of experiments to find the optimal number of Resblocks in the alignment and up-sampling blocks, respectively. We increased the Resblocks from 0 to 3 and 0 to 10, respectively. The number of parameters and the SR accuracy were proportional to the increase in the number of Resblocks, and the proposed DCAN achieved the best performance with three Resblocks in the alignment block and ten Resblocks in the up-sampling block. Figure 11 shows the PSNR result per iteration of the tool-off test on the number of Resblocks in the alignment and up-sampling blocks. The training was stable in each experiment. In Table 4, we present the optimal number of pixel-shuffle layers in the up-sampling block. We executed the pixel-shuffle layers 1 and 2. Therefore, the proposed DCAN performed best with two pixel-shuffle layers.

## 4. Experimental Results

### 4.1. Dataset

**Figure 12:**
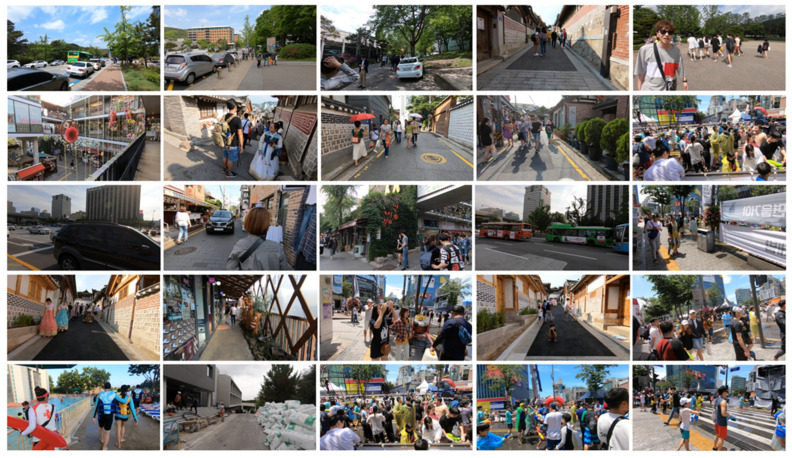
REDS training and test dataset.

**Figure 13:**
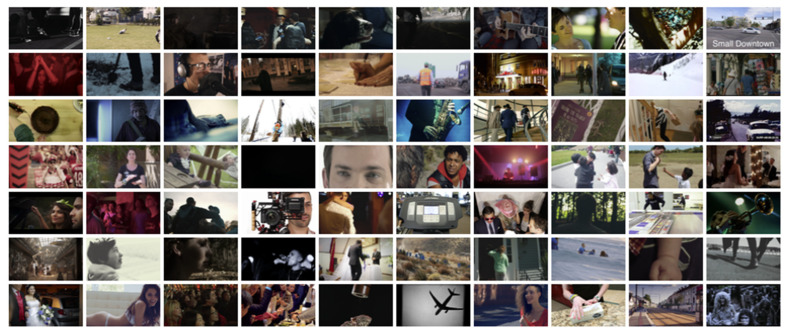
Vimeo-90K training and test dataset.

As shown in Figure 12 and Figure 13, we used realistic and dynamic sense (REDS) [53] and Vimeo-90K [54] video datasets. REDS consists of 240 training, 30 validation, and 4 test video clips, and each clip has 100 frames with a size of 1280 × 720. Vimeo-90K is composed of 91,701 training and 7824 test video clips (Vimeo-90K-T), and each clip has 7 consecutive frames with a size of 448 × 256. To collect the training data from REDS and Vimeo-90K, the training sequences were down-sampled using the bicubic method. The random patches were extracted with a size of 64 × 64.

### 4.2. Training of DCAN

Table 5 shows the hyperparameters to train the proposed DCAN. DCAN used L1 loss [55] as the loss function and the Adam [56] optimizer to update the kernel weights and biases. The batch size, number of iterations, and learning rate were set as 72, 10^−6^ to 10^−8^, and 500,000, respectively. The learning rate decay was 10^−1^, and the decay was reduced every 200,000 iterations. The training took approximately 4 days to complete.

All experiments were conducted on an Intel Xeon Gold 5220 (16 cores @ 2.20 GHz) with 256 GB RAM and three NVIDIA Tesla V100 GPUs under the experimental environment presented in Table 6.

In terms of SR performance, Table 7 and Table 8 show the results of PSNR and SSIM for the REDS4 and Vimeo-90K-T test datasets, respectively. We compared with the latest VSR methods such as TGA [48], SOF [49], TDAN [50], and STAN [51]. In Table 7, DCAN shows superior PSNR and SSIM compared to previous methods in the REDS4 test datasets. The proposed DCAN improved the average PSNR by 0.28, 0.79, 0.92, and 0.81 dB compared to STAN, TDAN, SOF, and TGA, respectively. DCAN improved SSIM gains by as high as 0.015, 0.025, 0.027, and 0.026, respectively. In the Vimeo-90K dataset, in Table 8, DCAN improved the average PSNR by 0.15, 0.67, 1.35, and 0.75 dB compared to the previous methods. DCAN also improved the average SSIM by 0.004, 0.008, 0.015, and 0.013, respectively. Therefore, the proposed DCAN outperformed the state-of-the-art STAN.

In terms of network complexity, we compared the number of parameters and total memory size with the compared methods. As shown in Table 9, DCAN reduced the number of parameters by 14.35% compared to STAN. Additionally, in Table 10, the proposed DCAN reduced the total memory by 3.29% compared to STAN. Table 11 shows that the proposed DCAN reduced the inference speed of the proposed method by 8.87% compared to STAN.

**Figure 14:**
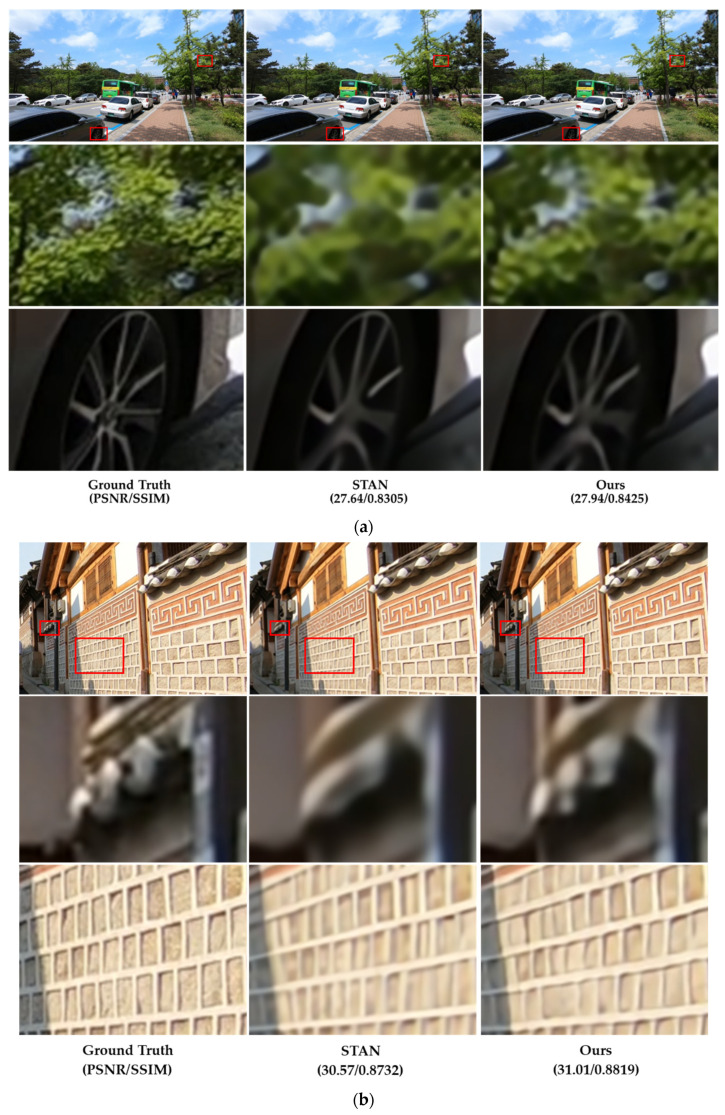

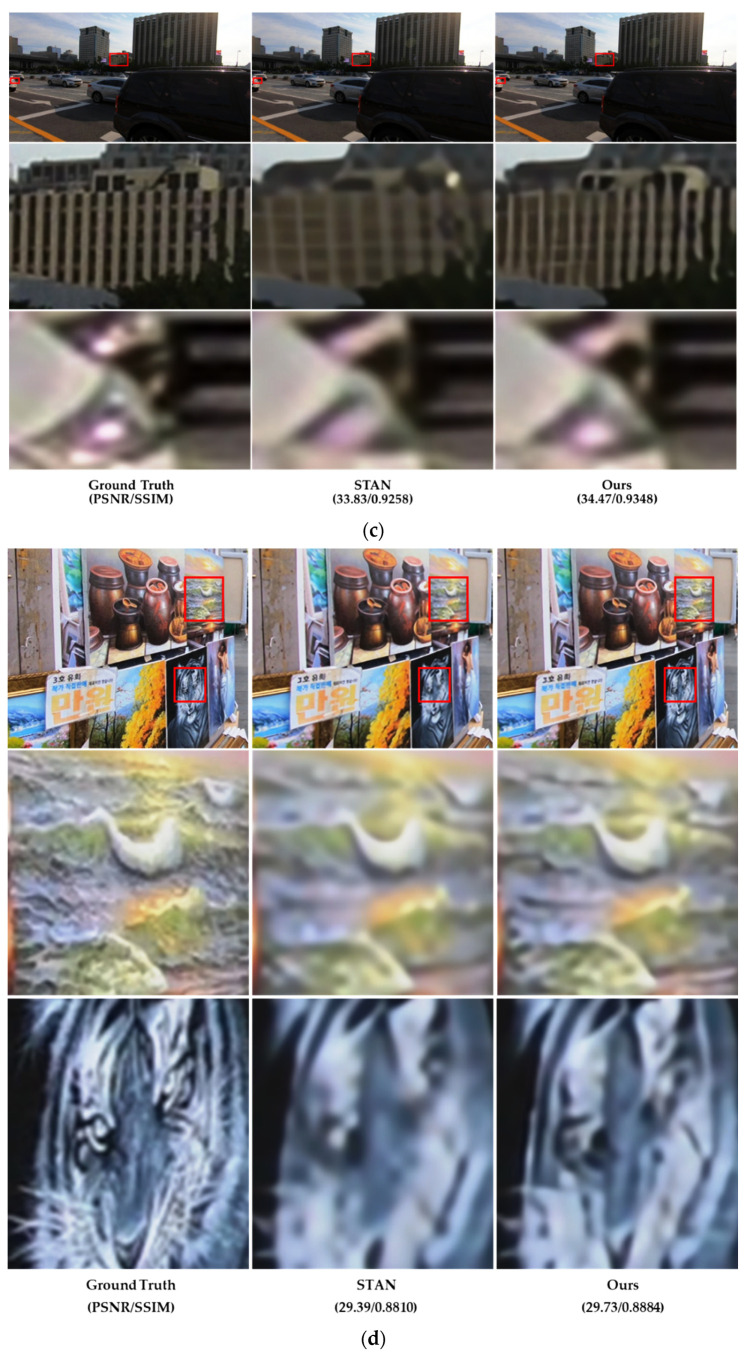
Visual comparisons on REDS4 test dataset ((**a**–**d**): clips 000, 011, 015, and 020 of the REDS training set). For a sophisticated comparison of test datasets, the figures of the second and third rows show the zoom-in for the area in the red boxes.

Figure 14 presents examples of visual comparisons between the proposed DCAN and STAN [51] on the REDS4 test datasets. Although STAN showed outstanding performance in the visual comparison with spatio-temporal learning, it had limitations in the high-frequency region. On the other hand, the proposed DCAN intensively found more accurate textures, and the edge region was expressed more conspicuously than STAN.

## 5. Conclusions

With the recent advances in sensor technology, image and video processing sensors have been used to handle the visual area. There is demand for high-quality and high-resolution images and videos. In this study, we proposed DCAN, which aims to achieve spatio-temporal learning through deformable-based feature map alignment. It generates HR video frames from LR video frames. DCAN is composed of FEB, alignment blocks, and an up-sampling block. We evaluated the performance of DCAN by training and testing with REDS and Vimeo-90K datasets. We performed ablation studies to determine the optimal network architecture considering AAB, DAB, and the number of Resblocks, respectively. DCAN improved the average PSNR by 0.28, 0.79, 0.92, and 0.81 dB compared to STAN, TDAN, SOF, and TGA, respectively. It reduced the number of parameters, total memory, and inference speed by as low as 14.35%, 3.29%, and 8.87%, respectively, compared to STAN.

To facilitate the use of sensors in lightweight memory devices with limitations of memory and computing environments, such as smartphones, methods to reduce network complexity are required. In the future, we aim to proceed with lightweight network research that can perform VSR in real-time.

## Figures and Tables

**Table 1:** Tool-off tests for the effectiveness of AAB and DAB. Each test result provides PSNR (dB), SSIM, and the number of parameters.

Model	Model 1	Model 2	Model 3	Model 4
AAB	×	×	⋎	⋎
DAB	×	⋎	×	⋎
PSNR↑	28.49	28.99	29.01	29.13
SSIM↑	0.8123	0.8316	0.8322	0.8354
No. of Parameters (×10^6^)	0.94	1.55	1.70	2.32

**Table 2:** Verification tests to determine the optimal number of Resblocks in the alignment block. Each test result shows PSNR (dB), SSIM, and the number of parameters.

No. of Resblocks in Alignment Block	0	1	2	3
PSNR↑	28.63	28.84	29.05	29.13
SSIM↑	0.8178	0.8264	0.8328	0.8354
No. of Parameters (×10^6^)	1.87	2.02	2.17	2.32

**Table 3:** Verification tests to determine the optimal number of Resblocks in the up-sampling block. Each test result provides PSNR (dB), SSIM, and the number of parameters.

No. of Resblocks in Up-Sampling Block	0	5	10
PSNR↑	28.40	28.84	29.13
SSIM↑	0.8139	0.8264	0.8354
No. of Parameters (×10^6^)	1.58	2.02	2.32

**Table 4:** Verification tests to determine the optimal number of pixel-shuffle layers. Each test result shows PSNR (dB), SSIM, and the number of parameters.

No. of Pixel-Shuffle Layers	1	2
PSNR↑	29.12	29.13
SSIM↑	0.8353	0.8354
No. of Parameters (×10^6^)	2.32	2.32

**Table 5:** Hyperparameters to train the proposed DCAN.

Hyperparameters	Options
Loss function	L1 loss
Optimizer	Adam
Batch size	72
Learning rate	10^−6^ to 10^−8^
No. of iterations	500,000
Initial weight	Xavier
Padding mode	Zero padding

**Table 6:** Experimental environment.

Experimental Environment	Options
Input size ($I_{LR}^{i}$)	64×64×3
Label size ($O_{HR}^{t}$)	256×256×3
Linux version	Ubuntu 18.04
CUDA version	11.3
Deep learning framework	Pytorch 1.11.0

**Table 7:** Average PSNR (dB) and SSIM on the REDS4 test datasets.

Network	PSNR↑(Delta)	SSIM↑(Delta)
TGA [48]	28.32 (−0.81)	0.8090 (−0.026)
SOF [49]	28.21 (−0.92)	0.8083 (−0.027)
TDAN [50]	28.34 (−0.79)	0.8106 (−0.025)
STAN [51]	28.85 (−0.28)	0.8207 (−0.015)
Ours	29.13	0.8354

**Table 8:** Average PSNR (dB) and SSIM on the Vimeo-90K-T test datasets.

Network	PSNR↑(Delta)	SSIM↑(Delta)
TGA [48]	33.48 (−0.75)	0.9074 (−0.013)
SOF [49]	32.88 (−1.35)	0.9045 (−0.015)
TDAN [50]	33.56 (−0.67)	0.9118 (−0.008)
STAN [51]	34.08 (−0.15)	0.9162 (−0.004)
Ours	34.23	0.9199

**Table 9:** Comparisons of the number of parameters.

Network	No. of Parameters (×10^6^)
TGA [48]	7.06
SOF [49]	1.64
TDAN [50]	1.97
STAN [51]	16.16
Ours	2.32

**Table 10:** Comparisons of the total memory size.

Network	Total Memory Size (MB)
STAN [51]	9390.54
Ours	309.26

**Table 11:** Comparisons of the inference speed on REDS4.

Network	Inference Speed (s)
STAN [51]	42.37
Ours	3.76

## Data Availability

Not applicable.
